# Appendectomy: Cross-sectional study of the effects of COVID-19 in a hospital in South Brazil

**DOI:** 10.1016/j.sopen.2024.08.003

**Published:** 2024-08-22

**Authors:** Tierre Aguiar Gonçales, Thiago Lucas Bastos de Melo Moszkowicz, Mariana Severo Debastiani, Marcos Souza Parreira, Julia Kasali Lima, Rafael José Vargas Alves, Claudia Giuliano Bica

**Affiliations:** a Graduate Program in Pathology at the Federal University of Health Sciences of Porto Alegre (UFCSPA), Porto Alegre, RS, Brazil; b Undergraduate Medicine Program at the Federal University of Health Sciences of Porto Alegre (UFCSPA), Porto Alegre, RS, Brazil; c Department of Internal Medicine, Federal University of Health of Porto Alegre (UFCSPA), Porto Alegre, RS, Brazil; d Department of Basic Health Sciences, Federal University of Health of Porto Alegre (UFCSPA), Porto Alegre, RS, Brazil; e Clinical Oncology Department, Hospital Santa Rita, Complexo Hospitalar Santa Casa de Misericórdia de Porto Alegre, Porto Alegre, Rio Grande do Sul, Brazil

**Keywords:** Acute appendicitis, Appendectomy, COVID-19, Emergency surgery, SARS-CoV-2

## Abstract

**Background:**

COVID-19 has further burdened the Brazilian healthcare system, especially emergencies. Patients may have delayed seeking care for surgical abdominal pain. Delays in the approach may have impacted clinical evolution and outcomes. This study evaluated appendectomies and their complications performed by the public system during one-year follow-up of COVID-19 in a hospital in southern Brazil.

**Materials and methods:**

In this hospital-based cross-sectional study, we included adult patients who underwent appendectomy from March 2019 to April 2021 (n = 162). Patients were divided into pre-pandemic (n = 78) and pandemic (n = 84) groups based on the surgery date. The analyzed variables included hospitalization duration, intensive care unit (ICU) admission, surgical approach, histopathological findings, COVID-19 testing, patient outcomes, and 30-day survival rate.

**Results:**

The cohorts exhibited similar epidemiology, with the sex ratio and average age being maintained. No statistical difference was found in the 30-day survival rate and clinical outcomes. Of the four patients admitted to the ICU, three belonged to the pandemic cohort and tested negative for COVID-19. Only 47.6 % of the patients in the pandemic cohort underwent COVID-19 polymerase chain reaction examination; one tested positive (2.5 %).

**Conclusion:**

This study demonstrated that there was no increased risk for appendectomies during the first wave of the pandemic. Surgeries were safe during this period. Patients continued to access the emergency service despite surgical abdominal pain and restrictive measures imposed by health authorities. The similar results observed across cohorts are attributed to the readiness of the teams and the availability of medical surgical equipment in safe quantities.

## Highlights

• First study in Brazil to evaluate appendectomy patients during one-year follow-up of the COVID-19 pandemic.
• The pandemic did not affect clinical results of appendectomies, even with the overburdenet Brazilian healthcare system.
• There was no increase in SARS-CoV-2 infection in patients indicated for appendectomy.
• Anatomopathological analysis did not show worst degree of appendicitis evolution in the first year of the pandemic.

## Introduction

Appendicitis is the leading cause of acute surgical abdomen, with a global prevalence of 7 %, and it requires surgical intervention, which is the gold standard treatment for this condition [1]. This condition is an abdominal emergency that requires surgery and is one of the most common reasons for non-traumatic hospital admissions. Owing to the diverse presentations of the condition, delays in diagnosis, which are crucial for patient management and outcomes, lead to more complex surgical procedures with a poorer prognosis, particularly in terms of postoperative morbidity and mortality. Delays in initiating treatment can lead to complicated appendicitis, which is marked by gangrene, abscess, perforation, or complete necrosis of the appendix [2,3]. In Brazil, the mortality rate for appendicitis is approximately 0.35 %. In 2020, there were 441 deaths, indicating an increase from the reported deaths in 2019 (411 deaths) [4].

The unprecedented crisis caused by the pandemic has further burdened the Brazilian healthcare system, particularly the emergency services [5,6]. Hospitals began to set up treatment regimens and limit resources as the virus spread [5,[7], [8], [9], [10]]. Emergency measures were implemented to prioritize the allocation of material and human resources and to protect the health of frontline workers [5,6,8,10]. During this initial period of severe restrictions, there were concerns regarding the clinical outcomes of patients with appendicitis, as delays in surgical intervention could lead to higher complication rates and an increased risk of severe acute respiratory syndrome coronavirus 2 (SARS-CoV-2) infection [11]. In this context, patients may have delayed seeking hospital care even when experiencing surgical abdominal pain [12,13]. Non-surgical treatment could be a safe alternative to surgery in early cases [[14], [15], [16]]. In some areas, there was a significant reduction in emergency surgeries and greater interval between the onset of symptoms and surgical intervention [17,18]. In these cases, increased morbidity rates were observed, but there was no statistical difference in the mortality rates or the need for reoperation [12,19].

In southern Brazil, the situation mirrored others, with the first case of COVID-19 reported in March 2020. Within 1 year, the region experienced its peak of infections, marking the first wave of the pandemic [20]. To curb the viral spread, authorities implemented restrictive measures, shutting down non-essential services and advising individuals to seek emergency care only if they exhibited severe symptoms. Elective surgeries were halted owing to the public health system's overload, particularly because of shortages in healthcare personnel, hospital beds, and surgical supplies [7,10]. This issue was even more critical concerning intensive care unit (ICU) beds [5]. However, emergency surgeries were still performed [10]. In this context, given the restrictive measures imposed by the pandemic, it is important to analyze the impact that this decision had on the clinical outcomes of patients who underwent appendectomy. Therefore, in this study, we aimed to assess and to evaluate appendectomies performed by the SUS and their complications during the first wave of the COVID-19 pandemic in a tertiary hospital in southern Brazil.

## Materials and methods

### Study design and patient profile

In this hospital-based cross-sectional study, we included adult patients who underwent appendectomy from the Government Health System (SUS) admitted to a Complex that comprises 9 hospitals of the Irmandade da Santa Casa de Misericórdia de Porto Alegre (ISCMPA) in Southern Brazil between March 1, 2019 and April 30, 2021, with a 30-day follow-up post- discharge period. The pre-pandemic and pandemic cohorts comprised individuals that underwent surgery from March 1, 2019 to March 31, 2020 and from April 1, 2020 to April 30, 2021, respectively. In the State of Rio Grande do Sul alone, 548 appendix surgeries were performed by the Public Health System (SUS) during the first peak of the pandemic. At ISCMPA, a reference in state public and private care, in the period comprising the first year of the pandemic, 281 appendix surgeries were performed by the SUS [21].

The variables were collected through the institutional electronic medical record system (Tasy®, Phillips Healthcare, Amsterdam, The Netherlands), with REDCap (Research Electronic Data Capture) serving as the data collection tool used by a team trained by the lead researcher. The descriptive variables examined included the hospitalization duration (the duration from admission to discharge), postoperative complications, 30-day outcomes (the time from admission to 30 days post-discharge), reverse transcription polymerase chain reaction (RT-PCR) test (pandemic cohort), clinical outcome, surgical approach, and sociodemographic and clinicopathological variables. The classification of appendicitis was based on the evolutionary stage, and confirmed by a pathologist. Uncomplicated appendicitis refers to stages I or II, whereas complicated appendicitis refers to stages III or IV [22]. The Strengthening the Reporting of Observational Studies in Epidemiology (STROBE) guidelines were used to guide the writing of this study [23].

The study received approval from the appropriate Institutional Review Board (approval number: 5.354.532). The study adhered to the tenets of the Helsinki Declaration and maintained data confidentiality in accordance with the General Data Protection Law, safeguarding each individual's fundamental rights to freedom and privacy.

The primary outcome was the clinical endpoint. The secondary outcome pertains to the extent of appendicitis progression, as confirmed by histopathological examination. Surgical complications requiring ICU admission were categorized as Grade IV on the Clavien–Dindo scale [24].

### Statistical analysis

Qualitative variables are reported as absolute and relative frequencies, while quantitative variables are expressed as means and standard deviations or medians and interquartile ranges, along with absolute and relative frequencies, as appropriate (n = 162). The association analysis between the pre-pandemic and pandemic cohorts with continuous age was assessed using the independent *t*-test; for the length of hospital stay, the Mann–Whitney *U* test was used, and for other qualitative factors, the chi-square test of association was used. All analyses were conducted using SPSS software version X (IBM Corp., Armonk, NY, USA), with the significance level set at 5 %.

## Results

**Fig. 1:**
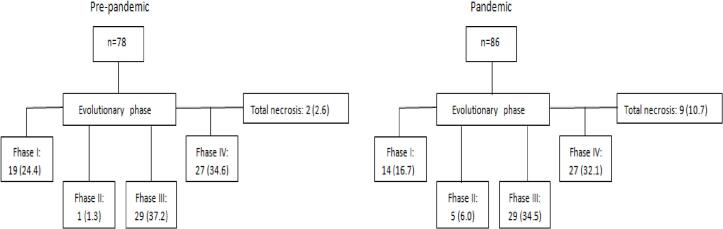
Study profile distributed according to degree of anatomopathological evolution.

In the period from March 1, 2019 to March 31, 2020, pre-pandemic, 281 appendectomies were performed by the public health system, of which 78 were included in the study. In the pandemic cohort, from April 1, 2020 to April 30, 2021, 237 appendix surgeries were performed, with 84 patients included in the study. The patient flowchart and the evolutionary phase of each group are presented in Fig. 1.

As shown in Table 1, our findings revealed no statistically significant differences concerning demographic characteristics, clinical outcomes, in-hospital mortality rates, 30-day mortality rates, and ICU admissions. The average age of patients was 37.4 years. In the pre- pandemic cohort, the average age was 38.0 years, whereas during the pandemic, it was 36.9 years (*p* = 0.669). Of the 162 patients analyzed, 139 (85.8 %) were aged <60 years. In the pre-pandemic period, this age group accounted for 82.1 %, whereas during the pandemic, it represented 89.3 % of the cohort (*p* = 0.187). The age distribution within the cohorts showed no significant variation. However, fewer older individuals underwent appendectomy during the first wave of the pandemic, than during the same pre-pandemic period (10.7 % vs. 17.9 %, respectively, *p* = 0.187). Before the pandemic, there were no recorded hospital or 30-day deaths, while there was only one ICU admission. During the pandemic, there were two deaths recorded during hospitalization (2.4 %), one death within 30 days (1.2 %), and only four ICU admissions (4.8 %). However, the statistical analysis results indicated that there was no significant difference in these data.

**Table 1:** Baseline clinical characteristics of all patients, pre-pandemic cohort compared to the pandemic cohort.

Characteristic	Total n = 162	Pre-pandemic n = 78	Pandemic n = 84	Confidence interval 95 %	*p*
Age (average and standard deviation)	37.4 ± 16.4	38.0 ± 16.8	36.9 ± 16.2	−1.2 (−6.3–4.0)	0.669^a^
Age					
<60 years	139 (85.8)	64 (82.1)	75 (89.3)	7.2 (−3.6–18.4)	0.187^b^
Sex					
Male	88 (54.3)	40 (51.3)	48 (57.1)	5.9 (−9.5–21.2)	0.454^b^
Hospital death	2 (1.2)	0	2 (2.4)	2.4 (−0.9-5.6)	0.497^b^
Death within 30 days	1 (0.6)	0	1 (1.2)	1.2 (1.1–3.5)	>0.999^b^
ICU admission	5 (3.1)	1 (1.3)	4 (4.8)	3.5 (−1.7–8.7)	0.368^b^
Surgical access					
Conventional	15 (9.3)	6 (7.7)	9 (10.7)	3 (−5.9–11.9)	0.507^b^
Videolaparoscopy	147 (90.7)	72 (92.3)	75 (89.3)		
Length of stay (median [IQR])	2 [2–4]	3 [2–4]	2 [2–4]		0.514^c^
COVID-19					
RT-PCR emergency service		–	40 (47.6)		
RT-PCR positive		–	1 (2.5)		
Classification (evolutionary phase according to AP)					0.119^b^
Uncomplicated (phases I and II)	39 (24.1)	20 (25.6)	19 (22.6)		
Complicated (phases III and IV) and NTa	123 (75.9)	58 (74.4)	65 (77.4)		0.653^b^

Abbreviations: AP, anatomopathological; IQR, interquartile range; PCR, polymerase chain reaction; TNA, total necrosis of the appendix.

a Independent samples *t*-test.

b Chi-square test of association.

c Mann–Whitney *U* test.

Regarding the surgical approach, before the pandemic, six (7.7 %) patients underwent conventional surgery, whereas during the pandemic, nine (10.7 %) cases were recorded (*p* = 0.507). There was no statistically significant association between the cohorts and the pathological findings (*p* = 0.119). However, as shown in Table 1, there was a specific increase of 3 % (from 74.4 % to 77.4 %) in the incidence of complicated appendicitis and a decrease of 3 % (from 25.6 % to 22.6 %) in non-complicated appendicitis cases. A normal appendix was not reported in any pathological report (stage 0). In 11 cases, anatomopathological analysis was not performed (2.6 % vs. 10.7 %, *p* = 0.105). In that regard the diagnosis was confirmed during surgery, corroborating the surgeon's assessment, and was described in the pathological report as complete necrosis of the appendix. Regarding the number of appendectomies and the evolution of appendicitis, the data were similar in both cohorts.

In comparison, regarding hospitalization duration and its association with complicated appendicitis, there was no significant difference between the pre-pandemic and pandemic cohorts (*p* = 0.514 and *p* = 0.119, respectively). However, among patients hospitalized during the pandemic, of the 84 patients, only 40 (47.6 %) underwent a RT-PCR test for COVID-19 upon arrival at the emergency department. Among these, only one (2.5 %) has tested positive for SARS-CoV-2.

## Discussion

To the best of our knowledge, this is the first study conducted in Brazil that reported that there was no increased risk for appendix surgeries associated with the first wave of COVID-19. The anatomopathological evaluation confirmed that there was no worsening in the evolution of appendicitis during the studied period. In other words, even with the public health system operating at the limit of its capacity, it was possible to keep the general mortality rate related to acute appendicitis unchanged during the dark period of our current history.

Our study showed that there was no statistically significant difference in demographics, clinical outcome, hospital and 30-day mortality and ICU admission. However, during the first wave of the pandemic, elderly people were the most likely to undergo appendectomy. During this period, two deaths were recorded during hospitalization and one death within 30 days of hospital discharge, as well as three times more ICU admissions, but without statistical significance.

A single-center study conducted in a developed country showed that the appendectomy postoperative complications, reoperations, and readmissions were significantly higher during the pandemic than those before [11]. Numerous studies have indicated that governmental policies and the impacts of the pandemic led to varied outcomes, especially concerning the incidence and postoperative results of appendicitis worldwide [5,[25], [26], [27], [28], [29], [30], [31]]. In an effort to conserve hospital beds, medications and reduce SARS-CoV-2 infections, Brazilian authorities imposed mobility restrictions on the population and advised states to postpone elective surgeries in March 2020 [[7], [8], [9]]. Consequently, surgical services had to be adapted, prioritizing emergency operations and reinforcing the optimisation of available resources. We were facing a novel disease with no established treatment or prognosis and without scientifically proven measures to contain viral transmission. Only in January of the following year, Brazil began its vaccination campaign, prioritizing the older individuals and healthcare professionals.

In March 2021, the month that witnessed the highest mortality rate in southern Brazil, the Gamma variant emerged with a lethality rate of 3.8 %, and only 8.4 % of the regional population had been vaccinated [32]. As a result of the suspension of elective surgeries, ward and ICU beds become available, thereby increasing the bed capacity for patients infected with the virus. This action also freed up surgeons to assist in emergency care. Despite the more contagious strain and considering that patients with appendicitis undergoing surgical procedures experience compromised immunity, all exhibited a good outcome. Therefore, the hospital's sanitary measures for viral containment were sufficient to achieve these results.

Interestingly, a study observed a statistically significant reduction in appendicitis cases during the first wave of the pandemic compared to those observed in the same period of the previous year [12,33]. However, several authors have reported an increase in complicated appendicitis cases during the first wave [12,13,27,31,34,35]. Reinforcing the findings from our service, some authors have reported that patients with emergency surgical conditions continued to access the healthcare system, with no statistical difference between the diagnosis and progression of appendicitis [[36], [37], [38], [39]]. The clinical presentation and stage of progression are independent predictors of postoperative outcomes [11]. Experiences from developed countries during the first wave showed similar results regarding clinical outcomes when compared to those observed in the same period of the previous year [28,[40], [41], [42], [43], [44]]. Despite fears of contamination and mobility restrictions on the population, patients in the southern regions did not hesitate to seek hospital care when experiencing acute abdominal symptoms. Healthcare teams did not delay surgical intervention, resulting in similar outcomes concerning patient recovery and the number of surgeries performed.

Although the frontline workers wore personal protective equipment, considering the early stages of the pandemic and swift clinical decline of those infected, the workers were widely infected [6]. This led to increased strain on surgical services owing to the reduced availability of these specialists because of infection or quarantine. Teixeira et al. reported that the pandemic had a negative impact on the physical and mental health of these workers [6]. Given the looming risk of healthcare services being overwhelmed, there was a need for organization in terms of resources and to ensure the safety of patients undergoing surgery [5,6].

Evidence on the management of appendicitis during the first wave is limited. Many of these recommendations are grounded in expert opinions [26]. In this context, a multicentric study encompassing 66 countries examined the management of appendicitis during the early phase of the COVID-19 pandemic [45]. While elective surgical procedures may be deferred, appendectomy interventions are typically urgent and cannot be delayed in most instances. Despite facing a shortage of professionals, exhausted and emotionally strained teams, a lack of hospital supplies, and a limited number of ward and ICU beds, there was no delay in surgical referrals or difference in morbidity and mortality rates in our service. The protocols implemented during the pandemic proved to be effective, making it unnecessary to postpone the operation. Emergency surgeries shouldn't be postponed due to delays in test results.

On the other hand, the services that opted for non-surgical treatment were more likely to have long-term complications or need for subsequent surgical intervention. Almost half of the patients in the pandemic cohort were tested for the virus, during screening, in the emergency department, characterizing community infection. Since the second half of 2020, the RT-PCR test has been recommended as the gold standard for hospitalized patients or those who may require surgical intervention [7].

Emergency surgeries should not be postponed due to delays in test results [9]. In July of the following year, it was advised to screen individuals in contact with healthcare services, and to isolate the suspected and confirmed cases [7]. In other countries, depending on the situation, it was considered reasonable to postpone appendicitis surgeries in their early stages because of the lack of available beds and medical-surgical supplies, or until a negative test result was obtained. All these reasons could have increased the incidence of complicated appendicitis [27,37,46].

However, even following the recommendations of the health authorities, and with the dedication of the health teams in carrying out screening tests for SARS-CoV-2 in our service, such measures were not important in postponing appendectomies. Meanwhile, in patients with positive results, there was a greater focus on the general functionality of the system, the protection of healthcare workers from exposure and the guarantee of the appropriate use of personal protective equipment, with a preference for traditional surgical methods.

### Limitations

This study has some limitations. First, it examined cases of appendix surgeries performed during one-year follow-up of the COVID-19 pandemic and had a single-center retrospective design. However, it was precisely this made it possible to examine this singular time of the pandemic. During the period when movement restrictions and lockdowns were enforced, there was widespread panic among the population because of the risk of contamination in emergencies, the unprecedented nature of the disease, and, most importantly, the lack of scientifically proven preventive measures. Another limiting factor was the exclusion of patients who underwent conservative treatment, resulting in selection bias. Additionally, the costs of medical-surgical supplies in the pandemic were not assessed, as these expenses would be incomparable to pre-pandemic, due to the huge increase in market price during this time.

## Conclusions

We can see that even with the public health system facing an imminent shortage of human and material resources for decades, it was possible to keep it functioning so that Brazilian lives could be spared with adequate surgical assistance for appendicitis. Unlike the rest, developed countries, the pandemic did not change the status of appendicitis. In other words, there was no worsening of morbidity and no increase in mortality during this dark period in our history.

With the data presented, we can corroborate that the health system was not only able to quickly organize itself in the face of the global crisis, but also maintain quality care, saving lives. Pathological analyzes of the appendages also confirmed, unlike other countries, that in the South of Brazil there was no worsening in the evolutionary phase of the disease in relation to the previous year.

These results indicate that we can maintain and improve public health measures in the face of other pandemics and, mainly, the conduct adopted in our institution such as the use of Personal Protective Equipment, the cancellation of elective surgeries (saving surgeons and health professionals, saving medicines, infirmary beds, ICU beds, for example) and the training of more than professionals to work on the front line.

Surgeons can indicate surgical treatment of appendicitis safely during a SARS-CoV-2 pandemic as long as there is a compatible clinical indication and, mainly, available resources, materials and personnel. Although these findings are constrained by the analysis of a single center and one type of surgical pathology, they support the overall adaptability and sufficiency of the Brazilian healthcare system, which did not affect the morbidity and mortality rates. The resilience of frontline workers and the availability of equipment may account for the similar outcomes. It is important to conduct more studies to examine the effects of immunization on patients that underwent emergency surgery, especially during the period after the first year of the pandemic.

## Funding

The authors have not disclosed any specific grant for this research from funding agencies in the public, commercial, or not-for-profit sectors.

## Provenance and peer review

Not ordered.

## Ethics approval

The study received approval from the appropriate Institutional Review Board (approval number: 5.354.532).

## CRediT authorship contribution statement

**Tierre Aguiar Gonçales:** Writing – review & editing, Writing – original draft, Visualization, Validation, Supervision, Project administration, Methodology, Investigation, Formal analysis, Data curation, Conceptualization. **Thiago Lucas Bastos de Melo Moszkowicz:** Writing – review & editing, Writing – original draft, Visualization, Validation, Supervision, Project administration, Methodology, Investigation, Formal analysis, Data curation, Conceptualization. **Mariana Severo Debastiani:** Writing – original draft, Visualization, Validation, Supervision, Project administration, Methodology, Investigation, Formal analysis, Data curation, Conceptualization. **Marcos Souza Parreira:** Writing – original draft, Visualization, Project administration, Methodology, Investigation, Formal analysis, Data curation, Conceptualization. **Julia Kasali Lima:** Writing – original draft, Visualization, Validation, Project administration, Methodology, Investigation, Formal analysis, Data curation, Conceptualization. **Rafael José Vargas Alves:** Writing – review & editing, Writing – original draft, Visualization, Validation, Supervision, Project administration, Methodology, Investigation, Formal analysis, Data curation, Conceptualization. **Claudia Giuliano Bica:** Writing – review & editing, Writing – original draft, Visualization, Validation, Supervision, Project administration, Methodology, Investigation, Formal analysis, Data curation, Conceptualization.

## Declaration of competing interest

We attest that this manuscript has not been published elsewhere and is not under consideration by another journal. All authors have approved the manuscript and agree with submission to Surgery Open Science. The authors have no conflicts of interest to declare.
